# Factors behind healthy snack consumption at school among high-school students: a qualitative study

**DOI:** 10.1186/s12889-019-7656-6

**Published:** 2019-10-22

**Authors:** Fatemeh Bastami, Fereshteh Zamani-Alavijeh, Firoozeh Mostafavi

**Affiliations:** 1 Department of Public Health, School of Health and Nutrition, Lorestan University of Medical Sciences, Khorramabad, Iran; 2 Department of Health Education and Promotion, School of Health, Isfahan University of Medical Sciences, Isfahan, Iran

**Keywords:** Snack, Student, Qualitative study

## Abstract

**Background:**

The prevalence of consuming fast foods and non-nutritious snacks is progressively increasing among adolescents. This study aimed to explore factors behind snack consumption at school among Iranian high-school students.

**Methods:**

This descriptive qualitative study was conducted in 2017 in four boys’ and four girls’ high-schools located in Isfahan, Khorramabad, and Tehran, Iran. Data were collected through 42 in-depth semi-structured interviews and four focus groups with male and female students, their parents, and their school teachers and administrators. The conventional content analysis approach was used for data analysis. Trustworthiness was applied to the study through prolonged engagement, maximum variation sampling, and member checking techniques.

**Results:**

Factors behind students’ snack consumption came into two main groups, namely influential behaviors, and influential emotions and perceptions. Influential behaviors included the behaviors of students, their family members, peers, school administrators, and snack sellers. Moreover, influential emotions and perceptions included positive and negative feelings towards healthy snacks, fear over the consequences of unhealthy snacks, and perceived positive outcomes of healthy snacks.

**Conclusions:**

Students’ snack consumption at school is affected not only by their own behaviors, emotions, and perceptions, but also by significant others’ behaviors and environmental factors. School administrators need to make environmental modifications to turn school environment into a pleasant place for healthy snack consumption and make healthy snack consumption a pleasurable experience for students.

## Background

Snacking, defined as the intake of foodstuffs between main meals, is among the main sources of calorie intake. It accounts for around 40% of the total calorie consumed daily by Iranian school students [1, 2]. Around noontime, students, even those who eat breakfast, feel hungry and thus, lose concentration; therefore, an appropriate snack at this time can promote their concentration and learning [3].

Snacks should be chosen not only based on students’ interests and preferences, but also on their nutritional needs. Moreover, snacks should not affect their appetite for the main meals. Healthy snacks may include fresh fruits and vegetables, natural fruit juices, nuts (such as walnut, hazelnut, pistachio, and almond), and simple biscuits [3].

Studies in different areas of the world reported unhealthy snacking among students. For instance, a study in Poland found potato chips as the most commonly used non-nutritious snack among students [4]. Moreover, the most commonly used snacks among American adolescents were candies, carbonated drinks, sweet drinks, and high-salt snacks [3]. Studies in Iran also reported that children and adolescents eat unhealthy snacks [1, 2]. A systematic review in Iran also reported that the most commonly used unhealthy snacks in urban and rural areas were candies (30.8 and 33.2%), carbonated drinks (21.5 and 27.2%), and cheese puffs and potato chips (20.3 and 25.8%), respectively [5]. Consumption of unhealthy snacks can bring children and adolescents different health problems such as obesity, dental caries, and chronic illnesses. Moreover, consumption of these snacks during childhood and adolescence puts them at risk for developing health problems in adulthood such as cardiovascular diseases, hypertension, and diabetes mellitus [6].

Culturally speaking, acceptable snacks in Iran traditionally include wraps made from flatbread, cheese, tomatoes, and cucumbers, wraps made from flatbread, cheese, and walnut kernels, wraps made from flatbread, cheese, and herbs, and various kinds of *aash* (traditional Iranian pottage-like food) made from grains and pulses. However, as people’s lifestyles change and with industrialization, this is in transition towards the consumption of high-calorie and unhealthy foods, such as fast food. The factors contributing to the formation of both types of behaviors in Iran, the consumption of traditional foods and the consumption of fast food, need to be explored and explained. This issue and its related factors have not been adequately studied without prior assumptions and with a qualitative approach in the real context in which children and adolescents grow up, which could be why, despite previous studies and interventions children still adopt this unhealthy behavior [7–12]. The present study was conducted to explore factors behind snack consumption at school among Iranian high-school students.

## Methods

### Study design

This was a descriptive qualitative study. Qualitative studies are useful for exploring different aspects of poorly-known phenomena as experienced by people [13].

### Participants and settings

This study was conducted in four boys’ and four girls’ high-schools located in Isfahan, Khorramabad, and Tehran, Iran. Main participants or key informants were ten male and ten female high-school students with an age mean of fourteen. Besides these twenty main participants, we also recruited five mothers, five fathers, two school deputies, four teachers, four school health teachers, and two senior school health staff. Sampling was done purposively to recruit people with experiences about snack consumption. Sample size was determined based on data saturation [13].

### Data collection

Data were collected via 42 in-depth semi-structured interviews and four focus group discussions. Focus group discussions were held separately for male and female students and in six-person groups. Students who participated in focus group discussions were not the same as those who were personally interviewed. The questions were completely open-ended and there were no guidelines, and no prior theories or frameworks were used, because the conventional content analysis method was used, rather than the directed content analysis approach [14]. First, the differences between healthy snacks (such as healthy bites, nut and seed kernels, and *aash* (a sort of pottage) made from grains and pulses) and unhealthy snacks (like cheese puffs, potato chips, and fast food) were explained and clarified to them. Then, the two general questions that had been written on the instruction sheet for conducting the interviews and the focus group sessions were asked from them. Based on what they said, the subsequent questions were asked. Interviews were started using general questions such as “What factors make you eat healthy snacks?” “What factors make you eat unhealthy snacks?” Following these broad questions, exploratory questions were asked to collect detailed data on participants’ experiences. None of the participants were interviewed twice. Interviews and focus group discussions lasted 20–60 min, continued until reaching data saturation, and were recorded and immediately typed word by word. Interviews and focus group discussions in Khorramabad and Isfahan were conducted face-to-face; however, interviews with participants who lived in Tehran were conducted over the phone. None of the students refused participation, while some teachers in Khorramabad and Isfahan refused participation. Interviews and focus groups discussion with students and school staff were conducted at their schools, while parents were interviews at their homes.

### Data analysis

Data were analyzed through conventional content analysis in three phases, namely preparing, organizing, and reporting. In the preparing phase, each interview was considered as a unit of analysis and reviewed for several times for the purpose of immersion. In the organizing phase, initial-coding was used to organize the data. Accordingly, excerpts from the data which were related to factors behind students’ snack consumption were coded and after several interviews, the codes were grouped into subcategories. The same process was undergone for each interview. Further grouping of subcategories into main categories reduced the number of categories. Grouping was performed based on the similarities and differences among codes or subcategories. Finally, appropriate name were attached to the categories based on their content. Each of the authors independently performed data analysis. Then, all authors collectively examined the generated categories and agreed on their labels [14].

### Rigor

The first author was a PhD student and the others were associate professors. All authors were female and had previously done interventional studies on students’ health. They were interested in the study subject matter. Prolonged engagement in the study and spending adequate time for data collection helped us establish trust and rapport with participants and acquire detailed data. Moreover, sampling was performed with maximum variation to recruit participants with different characteristics from three large cities in Iran. This technique helped ensure transferability of the findings. In addition, member checking was performed with several participants to ensure the consistency between their experiences and the results of our data analysis. Necessary amendments were made to the findings based on their comments [13].

### Ethical considerations

This study was conducted after obtaining ethical approval from the Ethics Committee of Isfahan University of Medical Sciences, Isfahan, Iran (approval code: IR.MUI.REC.1396.3.420). Participants were informed about the study aim and the methods and were ensured that they could withdraw from the study at will. The place and the time of the interviews were determined based on participants’ preferences. Participants’ consent for participation in the study and for recording their voices was obtained both at the beginning of the study and during the interviews.

## Results

Initial codes were extracted from the transcriptions of the interviews and focus group discussions. These codes were classified into 15 subcategories, and 5 categories which were placed into two main groups. To avoid making the article too long, the initial codes have not been presented in a table. As an example, the initial code of “The collective consumption of healthy bites during the breakfast break” was placed in the subcategory of “School administrators’ behaviors”, which was place under the category of “Others’ behaviors affecting snack consumption”, which was itself subsumed under the main group of “Influential behaviors behind snack consumption”. Factors behind snack consumption at school were grouped into two main groups, namely influential behaviors with two main categories and influential emotions and perceptions with three main categories (Table 1).

**Table 1:** Factors behind high-school students’ snack consumption at school

Subcategories	Categories	Main categories
Breakfast consumption	Students’ own behaviors affecting snack consumption	Influential behaviors behind snack consumption
Snack preparation		
Staying up late		
Family members’ behaviors	Others’ behaviors affecting snack consumption	
Peers’ behaviors		
School administrators’ behaviors		
Snack sellers’ behaviors		
Tastelessness of healthy snacks	Positive and negative feelings towards snacks	Influential emotions and perceptions behind snack consumption
Misconceptions about snacks		
Fear over obesity	Fear over the consequences of unhealthy snacks	
Fear over illness		
Positive physical outcomes	Perceived the positive outcomes of healthy snacks	
Positive emotional outcomes		
Positive educational outcomes		
Positive social outcomes		

### Influential behaviors behind snack consumption

There were two types of influential behaviors behind snack consumption, i.e. students’ behaviors and others’ behaviors.

#### Students’ behaviors affecting snack consumption

Students’ behaviors which affected their snack consumption at school were grouped into the three subcategories of breakfast consumption, snack preparation, and staying up late.

##### Breakfast consumption

Breakfast consumption reduced the likelihood of consuming snacks at school. Contrarily, students who did not consume breakfast at home were more likely to consume unhealthy snacks at school. A student confirms, *“When I don’t eat breakfast, I eat sandwich at school”*.(A male student).

##### Snack preparation

Snack preparation was the second subcategory of students’ behaviors affecting their snack consumption. Some students prepared and took homemade snacks to school. A mother said, *“My child takes healthy snack from home to school and hence, avoids eating snacks such as puffs and chips at school”*. However, some students bought snacks from school café, fast food shops, or even peddlers. A school health teacher noted, *“Our students’ main problem is buying snacks from peddlers. If there were no peddlers, students did not bought from them”*.

##### Staying up late

Staying up late also affected students’ snack consumption because students who stayed up late were more likely to consume a non-nutritious snack at night. Late night snacks can reduce their appetite for breakfast, prevent them from consuming breakfast, and push them toward consuming unhealthy snacks at school. Students stayed up late mainly for either entertainment or studying. *“On holidays, I usually stay up late and play with my tablet. In these cases, I like to eat some junk foods even if I’m not hungry”*.(Group discussion 2, A female student).

#### Others’ behaviors affecting snack consumption

Family members, peers, school administrators, and snack sellers can also affect students’ snack consumption at school.

##### Family members’ behaviors

Family members, particularly parents, can act both as role models and supporters for healthy snack consumption. Moreover, they can involve students in preparing healthy snacks and encourage them to consume such snacks. A mother highlighted, *“One day, my daughter wanted to cook a food she had learned from a TV animation. I helped her to cook the food as a snack and served it to our family members. There, I encouraged her by telling family members that she had cooked the food. I think such practice can affect children’s ability to prepare and eat healthy foods”*.

##### Peers’ behaviors

Peers can also affect students’ snack consumption through both role-modeling for each other and social acceptance of each other. Peers with closer relationships are stronger role models for each other. *“If I buy warm snacks at school, my peers will also buy. They like me. We are five friends that are often with each other”*.(A male student).

Social acceptance, particularly during puberty, can also significantly determine snack consumption behaviors among students. They may succumb to do an activity in order to be socially accepted by their peers. A mother noted, *“My older son does not take homemade snack to school. Instead, he asks me money to buy something from school café. He also avoids eating fruits I put in his school bag because he says that his friends ridicule him”*.

##### School administrators’ behaviors

School administrators can also be good role models for snack consumption by students. They can help students learn healthy snack consumption through vicarious experience. A school health teacher mentioned, *“Teachers should eat warm healthy foods together with students. In this way, students will understand that warm foods served at school are healthy”*.

Moreover, school administrators can design and employ strategies for the promotion of healthy snack consumption by students. These strategies may include free milk distribution, provision of diverse healthy snacks at school café, consideration of an appropriate time and a clean and interesting place for eating healthy snacks, mandatory healthy snack consumption, and provision of educations about healthy snacks. Free milk distribution among students not only makes them consume a healthy snack, but also helps them develop healthy eating habits. A male student confirmed, *“They occasionally serve us milk at school. I previously was not accustomed to drink milk. But now, I am”*.(Group discussion 2).

Moreover, serving diverse healthy snacks at appropriate time and clean and interesting place prevents students from buying unhealthy snacks from fast food shops and peddlers and leads them towards eating healthy snacks. A female student said, *“We buy snacks from a nearby fast food shop because the foods in the school café are not diverse”*.

Mandatory healthy snack consumption is another strategy for the promotion of healthy snack consumption among students. A school health teacher said, *“Students should use the recommended healthy snacks. We have considered a healthy snack time, for which students are required to bring healthy snacks”*.

Healthy eating educations can also affect students’ snack consumption behaviors. A senior school health authority expressed, *“At the beginning of each school year, we provide all schools with the list of permissible and non-permissible snacks to be hanged at school café”*.

##### Snack sellers’ behaviors

Fast food sellers have pivotal roles in preparing and providing students with healthy or unhealthy snack foods. A participant highlighted, *“Fast food sellers use the oil ten times so that it finally turns into burned oil. Using burned oil in a car engine will break it down; so, what about human?”* (A male teacher).

### Influential emotions and perceptions behind snack consumption

Students’ snack consumption at school was also affected by their emotions and perceptions. The three categories of influential emotions and perceptions behind snack consumption are explained in what follows.

#### Positive and negative feelings towards snacks

Students may have negative or positive feelings towards different types of snacks. Such feelings can affect their snack consumption behaviors.

##### Tastelessness of healthy snacks

The taste of snacks was one of the most important factors behind our participants’ snack consumption behaviors. They believed that healthy snacks are not tasty enough and hence, had no positive feelings towards them. A male student said, *“Healthy ingredients are not important to me. Also, I don’t eat homemade snacks because I eat tasty snacks such as sandwich at school”*.(Group discussion 3).

##### Misconceptions about snacks

Students had also misconceptions about healthy and unhealthy eating which gave them positive or negative feelings about snacks. A female student stated, *“As snack, I need to eat some candies and avoid eating banana because banana has as much energy as a plate of rice”*.

#### Fear over the consequences of unhealthy snacks

Another main factor affecting snack consumption among school students was their fear over the consequences of eating unhealthy snacks.

##### Fear over obesity

Participants avoided eating non-homemade snacks due to the fear over obesity. They believed that while providing them with the necessary calorie, homemade snacks do not cause them obesity. A father said, *“There are cakes and fruit juices in school café; but, my daughter avoid eating them due to her fear over getting obese. Instead, she prefers to take homemade snacks such as bread and cheese to school”*.

##### Fear over illness

Participants had fear over using traditional snacks because they were prepared with hands in unsanitary conditions. Instead, they preferred non-nutritious snacks such as cheese puffs and potato chips which are produced using machines and hence, carry no risk of microbial infection. A female student confirmed, *“A snack which is produced by dirty hands contains microbes and can cause illness. For instance, I have seen hairs in bread and cheese snacks sold at school café. Therefore, I prefer packed puffs and chips to traditional bread and cheese because they are safer”*.(Group discussion 4).

#### Perceived positive outcomes of healthy snacks

Positive physical, emotional, educational, and social outcomes of healthy snacks required some participants to use them.

##### Positive physical outcomes

According to participants, healthy snacks can improve sleep quality and prevent overeating. A female student mentioned, *“We need to eat snack between lunch and dinner in order to eat lighter dinner and sleep better at night”*.

##### Positive emotional outcomes

Participants noted that preparation of healthy snacks at home has positive emotional outcomes for students. For instance, a mother said, *“My children say that their friends who don’t take healthy snacks have indifferent mothers”*.

##### Positive educational outcomes

According to participants, healthy snacks can promote students’ concentration and learning at school. A female student expressed, *“Difficult courses are taught at the first hours. They have considered a time for healthy snack eating in the first hours. Eating snack at this time supplies our brains with greater energy and helps us better understand lessons”*.

##### Positive social outcomes

Participants believed that failure to eat healthy snacks can negatively affect their social relationships. A male student confirmed, *“If you don’t eat healthy snack at school, your blood sugar falls, which causes boredom, aggression, and inability to establish good relationships with others”*.

## Discussion

This study explored factors behind snack consumption at school among high-school students. These factors were grouped into the two main groups of influential behaviors and influential emotions and perceptions. Influential behaviors included the behaviors of both students and others. Students’ behavioral habits such as eating breakfast or taking healthy snack from home to school are known factors behind healthy snack consumption at school [15]. However, engagement in sedentary activities such as watching TV or playing computer games has positive relationships with the consumption of non-nutritious snacks [16]. On the other hand, the behaviors of others such as family members, peers, and school staff can contribute to snack consumption among students. These individuals are role models for students and can determine their eating behaviors [17–19]. Because of peer pressure, peer acceptance, and greater desire for independence, adolescents usually do not follow healthy eating recommendations and hence, have unhealthy eating behaviors [20–22]. Accordingly, imperative approaches such as restriction of fast food consumption can make adolescents show resistance and continue their unhealthy eating behaviors. Given the greater effectiveness of social factors compared with personal factors in determining adolescents’ eating behaviors [23, 24], it seems that vicarious experiences from peers, family members, and school staff should be used to modify students’ eating behaviors.

Study findings also showed that snack consumption by students was affected by school administrators’ strategies such as free milk distribution, provision of diverse healthy snacks at school café, consideration of an appropriate time and a clean and interesting place for eating healthy snacks, and provision of educations about healthy snacks. Moreover, students’ snack consumption at school was affected by the availability of fast food shops or peddlers near school. Two earlier studies also reported the same findings [25, 26]. Thus, school administrators need to create a healthy and interesting environment at school and provide students with healthy snack options in order to prevent them from buying unhealthy snacks from fast food sellers and to encourage them to consume healthy snacks and adopt healthy snack consumption behaviors [27]. Besides, eating snacks in certain circumstances such as parties can bring people great happiness. Therefore, school administrators can provide students with the opportunity of healthy eating in happy group activities to promote healthy snack consumption among them.

Influential emotions and perceptions were the second main group of factors behind students’ snack consumption at school. One factor in this group which prevented students from consuming healthy snacks was their negative feelings towards these snacks due to their tastelessness. Taste is one of the most important criteria for adolescents and young adults in choosing foods [28]. because they have certain age-related physiological and psychological motives which lead them towards consuming more tasty fast foods [29]. A significant factor behind taste-related attitudes is exposure to new foods early in life, so that a study showed that exposure to a new healthy food for 8–15 times can create great desire for it [30]. Therefore, adolescents’ healthy snack consumption can be promoted through frequently exposing them to healthy snacks. Our findings also showed that students had misconceptions about healthy and unhealthy snacks. Strong positive beliefs about the outcomes of a behavior significantly affect individuals’ intention to show that behavior [31]. Therefore, strategies are needed to correct students’ misconceptions about snacks and thereby, promote their healthy snack consumption.

We also found students’ fear over the consequences of unhealthy eating as a factor behind their snack consumption. However, such fear occasionally prevented them from eating traditional foods offered in school café. In the Health Belief Model, fear is conceptualized as perceived threat and is considered as a significant factor behind behaviors [32], so that it is considered as a significant predictor of eating behaviors [33]. Therefore, strategies for the promotion of healthy snack consumption need to be based on the perceived threats of the target population. On the other hand, the findings of the present study revealed the positive outcomes of healthy snack as another factor behind snack consumption. The positive physical and psychological effects of healthy foods and the negative physical and psychological effects of unhealthy foods can discourage unhealthy eating and promote healthy eating. For instance, physical problems after fast food consumption (such as facial acne) or its associated psychological problems (such as negative feelings due to facial acne) can reduce the likelihood of further fast food consumption [34].

## Conclusion

This study concludes that students’ snack consumption is affected by their behaviors, perceptions, and emotions, their family members’ and peers’ behaviors, and their school environment. In other words, students’ snack consumption is determined not only by personal and interpersonal factors, but also by environmental factors. Education and awareness about the benefits of healthy snacks and the serious short- and long-term consequences of unhealthy snacks can be effective strategies for promoting healthy snack consumption among students. Environmental modifications might also turn school environment into a pleasant place for healthy snack consumption and make snack consumption a pleasurable experience for students.

## Data Availability

The datasets used and/or analyzed during the current study are available from the corresponding author on a reasonable request.
